# Selinexor and the Selective Inhibition of Nuclear Export: A New Perspective on the Treatment of Sarcomas and Other Solid and Non-Solid Tumors

**DOI:** 10.3390/pharmaceutics13091522

**Published:** 2021-09-20

**Authors:** Antonella Lucia Marretta, Giuseppe Di Lorenzo, Dario Ribera, Lucia Cannella, Claudia von Arx, Alessandra Bracigliano, Ottavia Clemente, Roberto Tafuto, Antonio Pizzolorusso, Salvatore Tafuto

**Affiliations:** 1 Department of Clinical and Surgery Oncology Unit, University of Naples “Federico II”, Via S. Pansini 5, 80131 Naples, Italy; antomarretta@live.com; 2 Sarcomas and Rare Tumors Unit, Istituto Nazionale Tumori—IRCCS, Fondazione “G. Pascale”, 80131 Naples, Italy; giuseppedilorenzo10@gmail.com (G.D.L.); l.cannella@istitutotumori.na.it (L.C.); ottavia.clemente@istitutotumori.na.it (O.C.); a.pizzolorusso@istitutotumori.na.it (A.P.); s.tafuto@istitutotumori.na.it (S.T.); 3 Department of Breast and Thoracic Oncology, Division of Breast Medical Oncology, Istituto Nazionale Tumori—IRCCS, Fondazione “G. Pascale”, 80131 Naples, Italy; claudia.vonarx@istitutotumori.na.it; 4 Nuclear Medicine Unit, Istituto Nazionale Tumori—IRCCS, Fondazione “G. Pascale”, Via M. Semmola 53, 80131 Naples, Italy; a.bracigliano@istitutotumori.na.it; 5 Division of Neurosurgery, University of Naples “Federico II”, Via S. Pansini 5, 80131 Naples, Italy; rob.tafuto@gmail.com

**Keywords:** selinexor, XPO1, SINE, soft tissue sarcoma, metastatic cancer, multiple myeloma, leukemia, gynecologic cancer, target therapy

## Abstract

Nucleocytoplasmic transport has been found dysregulated in many types of cancer and is often described as a poor prognostic factor. Specifically, Exportin-1 (XPO1) has been found overexpressed in many tumors and has become an attractive target in molecular oncology and therapeutics development. The selective inhibitor of nuclear export, Selinexor, is one of the most scientifically interesting drugs that targets XPO1 in clinical development. In this review, we summarized the most relevant preclinical and clinical results achieved for non-solid tumors, sarcomas, and other kind of solid tumors.

## 1. Introduction

**Figure 1:**
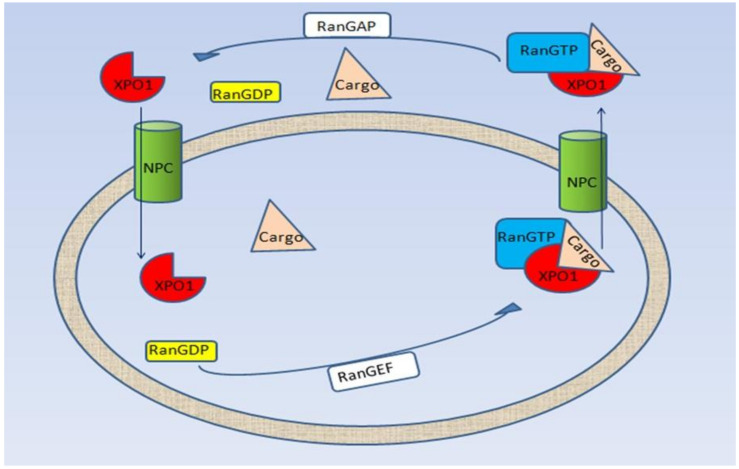
Schematic representation of the nucleocytoplasmic transport. The NPC * is composed by a complex structure of multiple rings. Cargo proteins (pink triangles in this figure) carriy an NES * bind to XPO1 * (red oval shapes in the figure) and RanGTP * (blue rectangles) through NPC (green cylinders). In the cytoplasm, hydrolysis of RanGTP to RanGDP * (by RanGAP *) allows complex dissociation. At this point, XPO1 can move back to the nucleus through NPC and the process resumes. * NPC: Nuclear Pore Complex; NES: Nuclear Export Signal; XPO1: Exportin-1; RanGTP: Ras-related Nuclear protein binding Guanosine 5′-TriPhosphate; RanGDP: Ras-related Nuclear protein binding Guanosine 5′-DiPhosphate; RanGAP: Ran-GTPase Activating Protein.

Nucleocytoplasmic transport is a mechanism of cellular homeostasis that is gaining increasing scientific relevance. In eukaryotic organisms, this process is essential for macromolecules transport through the nuclear membrane, thus contributing to cellular regulation. For molecules smaller than 40 kDa, the transport from the nucleus to the cytoplasm occurs through the Nuclear Pore Complex (NPC), a huge complex sited in the nuclear membrane and consisting of more than 30 different proteins called nucleoporin. For larger molecules import/export, transport is mediated by a group of Nuclear Transport Receptors (NTR), called importins and exportins, belonging to the karyopherin-β protein superfamily. Mechanistically, the importins facilitate the transport of cargo proteins into the nucleus through the binding of a specific domain rich in basic residue, known as Nuclear Localization signal (NLS). A similar approach is used by the exportins that transfer the cargo proteins outside the nucleus, but in this case the domain recognized on the cargo protein is rich in leucine and called Nuclear Export Signal (NES) [1]. The directionality of transport is regulated by the small GTPase Ran (Ras-related nuclear protein). Ran exists in both a GTP (Guanosine 5′-triphosphate) and GDP (Guanosine 5′-diphosphate) bound form [2]. In the nucleus, the Ran Guanine nucleotide Exchange Factor (RanGEF) elevates the nuclear concentration of RanGTP that has a high affinity for the exportins proteins and stabilizes the NES protein–exportin interaction. Thereby, the heterotrimeric complex NES protein–exportin–RanGTP can then diffuse to the cytoplasm where GTP is hydrolyzed by the Ran-GTPase Activating Protein (RanGAP) reducing the affinity of the exportins for the cargo molecules and thus releasing them. The export receptor returns empty into the nucleus for another round of export. By contrast, GTP displaces the NLS protein–importin interaction once the complex is in the nucleoplasm [3,4] (Figure 1). Nucleocytoplasmic transport has been found dysregulated in many types of cancer and is often described as a poor prognostic factor [5,6,7]. Specifically, the Exportin-1 (XPO1), also known as chromosomal region maintenance 1 (CRM1), a member of the karyopherin-β protein family, has been found overexpressed in many hematologic and non-hematologic malignancies. Of note, XPO1 overexpression correlates with poor clinical outcome in many kind of cancers (ovarian, pancreatic, osteosarcoma, glioma, and cervical cancer [8,9,10,11,12]. In recent studies, XPO1 has been identified as an export receptor for various proteins, as oncogenes as well as oncosuppressors, such as (p.53, [13]), BRCA1 [14], cyclin D1 [15], APC [16,17], forkhead box (FoxO) proteins [18,19], and topoisomerase I/II [20]. The changes in the localization of (proto-) oncoproteins and their deregulation are thought to be caused by the increase of cellular XPO1 levels, which ultimately influences (proto-) oncoproteins distribution. Considering the importance of proteins’ subcellular localization, it is not surprising that nucleocytoplasmic transport’s disruption is an oncogenic mechanism for cancer cells to evade and develop resistance to chemotherapeutic treatments. Given these findings, XPO1 has become an attractive target in molecular oncology and therapeutics development. The selective Inhibitor of Nuclear Export, Selinexor (KTP-330), is one of the most scientifically interesting drugs that targets XPO1 in clinical development. In this review, we analyzed Selinexor preclinical and clinical results of efficacy and safety, with a specific focus on its role in the treatment of soft tissue sarcoma.

## 2. Selective Inhibitor of Nuclear Export (SINE): Selinexor

**Figure 2:**
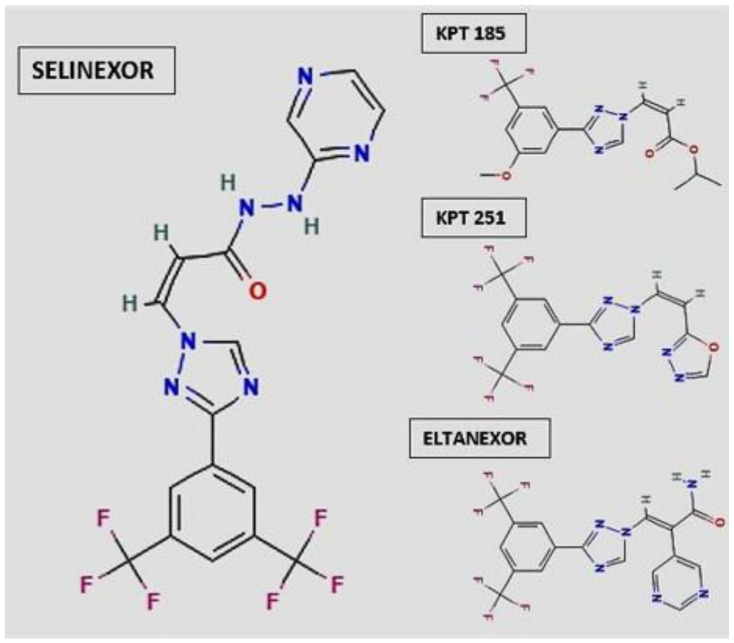
Selinexor, KPT185, KPT251, and Eltanexor chemical structures; in particular, Selinexor covalently binds to a cysteine residue in the nuclear export signal groove of human XPO1 [29].

Kalid O. et al., Negger J.E. et al. demonstrated how Selective Inhibitor of Nuclear Export (SINE) compounds bind to human XPO1, inhibiting the binding with NES cargo [21,22]. It has been proved that XPO1 inhibition locks cargo proteins, leading to selective apoptosis of cancer cells, whereas normal cells undergo transient cell cycle arrest [23,24]. In 2015, Gravina et al. studied the efficacy of six SINE analogues such as KPT-127, KPT-185, KPT-205, KPT-227, KPT251, and KPT-330, which share a trifluoromethyl phenyl triazole scaffold [25]. They evaluated the effects of these molecules in prostate cancer cells (PCa), showing that all the six SINE analogues can inhibit proliferation and promote apoptosis in PCa cells. Among the six SINE analogues, the KPT-127 was revealed to be the most potent in growth inhibition. Furthermore, Gravina et al., found a significant correlation between the efficacy of SINE compounds and XPO-1 protein expression levels, especially for KPT-127, KPT-207, KPT-330, and KPT-251 compounds. More recently, Sexton R. et al. analyzed the role of the second-generation SINE compounds KPT-8602 (Eltanexor) and KPT-185 in gastric cancer cell lines. As per the first generation compounds, KPT-8602 (Eltanexor) and KPT-185 both demonstrated strong efficacy in inhibiting cancer cell proliferation, by halting cell cycle progression at the G1/S phase and inducing apoptosis. Furthermore, they observed that the anti-tumor activity was concomitant with nuclear retention of tumor suppressor proteins and inhibition of colony formation [26]. In a recent phase I clinical trial, Abdul Razak et al., evaluated the safety and the efficacy of Selinexor [27]. In total, 189 patients with advanced solid tumors (in particular: 31% colorectal cancer, 11% head and neck squamous cell carcinoma, 11% prostate cancer, 8% melanoma, 6% pancreatic cancer, 33% other types of cancer) received Selinexor with a good toxicity profile; the most common grade 1 or 2 toxicities were fatigue (70%), nausea (70%), and anorexia (66%). Grade 3 or 4 toxicities were quite rare with the most common being thrombocytopenia (16%) and hyponatremia (13%). Therefore, the recommended phase II dose of 35 mg/m^2^ given twice a week was chosen according to patient tolerability and the fact that there was no difference in radiologic response or disease stabilization between the chosen dose and a higher dose. Thanks to these results, the Food and Drug Administration (FDA) has already approved Xpovio (Selinexor) in combination with dexamethasone, for the treatment of adult patients with relapsed refractory multiple myeloma [28]. Selinexor (KPT-330) became the first-in-class oral SINE compound currently being evaluated in multiple late-stage clinical trials in patients with relapsed and/or refractory solid tumor (Figure 2).

## 3. Pre-Clinical Studies

Selinexor has been evaluated in several pre-clinical studies on various cancer types. In the following paragraphs are presented some of the most relevant results.

### 3.1. Lung Cancer

Selinexor offers an interesting possible therapeutic strategy for non-small cell lung cancer (NSCLC), in fact one study showed in-vitro and in-vivo effects of Selinexor, using a large panel of 11 NSCLC cell lines containing different key driver mutations (for example: TP53, EGFR, PIK3CA). The antiproliferative activity of Selinexor was examined using MTT assay. The drug induced a dose-dependent growth inhibition after 72 h of exposure. On one hand, Western blot analysis showed a dose-dependent decrease in XPO1 levels, on the other, significant change was not observed in mRNA levels. Quite relevant in our opinion is the observation that the combination of KPT-330 with cisplatin synergistically enhanced NSCLC cells death in-vitro [30]. Based on these pre-clinical studies results, a phase I/II single-arm, non-blinded, multi-institutional clinical trial is ongoing to evaluate the safety and efficacy of Selinexor administered once weekly, adding concomitant docetaxel from the second week of treatment, in previously treated KRAS-mutant NSCLC [31].

### 3.2. Breast Cancer

Selinexor has shown promising results in pre-clinical studies in triple-negative breast cancer (TNBC). Survivin is a protein that acts as an oncogene when expressed in the cytoplasm; its transcription is promoted by STAT3 phosphorylation. Yan Cheng et al. demonstrated that Selinexor increases the nuclear accumulation of survivin and blocks STAT3 binding to survivin promoter. The overall effect results in decreased cytoplasmic levels of survivin and the promotion of cell death. Moreover, to determine the effects of XPO1 inhibition on TNBC growth in vivo, they used a human xenograft in SCID mice. Mice were divided into six different groups (8–10 mice/group) and treated with different doses of Selinexor (5–25 mg/kg/dose per os), 5-FU (40 mg/kg/dose i.p.) or vehicle alone for 42 days. Statistically significant differences were observed in tumor growth in mice treated with vehicle or 5-FU versus mice treated with Selinexor at 5 mg/kg 3 days a week (*p* = 0.020), Selinexor at 15 mg/kg 3 days a week (*p* = 0.011), Selinexor at 25 mg/kg 3 days a week (*p* = 0.008), and Selinexor at 25 mg/kg twice a week (*p* = 0.011). The data suggest that Selinexor induces a more than two-fold reduction in breast tumors growth when compared with vehicle alone or standard treatment with 5-FU [32].

### 3.3. Pancreatic Cancer

SINEs are largely investigated in the treatment of pancreatic cancer. One study demonstrated that SINEs inhibit proliferation and promote apoptosis of pancreatic cancer cells both in-vitro and in-vivo. This study focused on PAR-4 (prostate apoptosis response-4). In normal cells, ectopic PAR-4 is localized predominantly in the cytoplasm and does not induce apoptosis unless a second apoptotic insult occurs. In many cancers, PAR-4 has been found to be down-regulated; the nuclear localization of PAR-4 can lead to significant apoptosis in pancreatic cancer cells. The authors proposed PAR-4 activation as a potential therapeutic target in pancreatic cancer. Moreover, analysis of tumor remnants showed that Selinexor disrupts the interaction between XPO1 and PAR-4, activated PAR-4 signaling, and reduces the proliferation of tumor cells. Furthermore, oral administration of Selinexor to mice reduces the growth of subcutaneous and orthotopic xenograft tumors without major toxicity [33]. In another pre-clinical study, Selinexor, in combination with gemcitabine, demonstrated synergistic inhibition in pancreatic cancer cells; Sabiha Kazim et al. showed that Selinexor and gemcitabine promote apoptosis, induce p27, deplete survivin, and inhibit the accumulation of DNA repair proteins. Both Selinexor and Gemcitabine, when given as monotherapy, inhibit in a dose-dependent manner pancreatic cancer cells anchorage-dependent growth in-vitro and pancreatic tumor volume in-vivo; however, when Selinexor and Gemcitabine are administrated in combination, this effect is synergistically potentiated [34].

### 3.4. Melanoma

Since 2013, Dabrafenib and Trametinb have proven to be a revolution for melanoma treatment; these drugs inhibits BRAF and MEK pathways and their use has become an important strategy to treat those forms of melanoma that present a mutation of BRAF (V600E). Immunotherapy also continues to be important in the treatment of melanoma with the combination of more than one immunotherapeutic agent being the standard of care nowadays. Resistance to these novel therapeutic strategies may occur, with few active treatment available. Regardless of BRAF mutation status, it has been demonstrated that SINEs have a pro-apoptotic effects in melanoma cell lines; in fact, inhibition of ERK phosphorylation and nuclear localization of p53 are the most common mechanisms for tumor cells apoptosis with G1/S phase cell cycle arrest. Fragomeni et al. found that XPO1 inhibition decreases melanoma cell proliferation, and this decrease is independent from BRAF mutation status. In this study, authors observed that, 24 h after treatment, XPO1 inhibitors caused a reduction of S-phase and, subsequently, cell cycle arrest. Moreover, there are pieces of evidence of potential synergy between XPO1 and BRAF inhibition in BRAF-mutant melanoma [35], but this needs to be further investigated in a clinical setting.

### 3.5. Renal Cell Cancer

Fewer studies on SINEs are available in renal cell cancer (RCC), but published data demonstrate growth inhibition achieved by SINE compounds through the increase of nuclear localization of p21 and p53 that ultimately induces cell apoptosis both in-vitro and in-vivo. In a pre-clinical study, RCC cells were treated with Selinexor and cell cycle analyses were performed to evaluate the efficacy of Selinexor in two RCC cell lines. To show mechanisms of XPO1, immunoblotting and immunofluorescence analysis were performed. Moreover, Selinexor efficacy was observed in vivo, with RCC xenograft mice: There was RCC viability attenuation through growth inhibition and apoptosis induction, a process in which increased nuclear localization of p21 by XPO1 inhibition played a major role. Wettersten H.I. et al. demonstrated that many drugs used for RCC treatment like Sunitinib and Sorafenib, multikinase inhibitors, and Everolimus, an mTOR inhibitor, can be useful for Selinexor resistant cells. In vivo, Selinexor efficacy is similar to that of multikinase inhibitors, and for this reason, Selinexor represents a possible future target therapy [36].

### 3.6. Glioblastoma Multiforme

Glioblastoma (GBM) standard medical treatment is a temozolomide-based therapy. However, GBM is poorly responsive to the chemotherapy and the treatment options for the disease are lacking. In different studies, GBM showed a high expression of the nuclear transporter exportin 1 (XPO1, CRM1), considered predictive of a poor prognosis. To demonstrate the efficacy of SINE analogues in GBM, three different SINE compounds (KPT-251, KPT-276, and Selinexor) were tested in neurosphere cultures from seven patient-derived GBM cells. The authors found a dose-responsive growth inhibition in all GBM cell lines. Furthermore, KPT-251, KPT-276, and Selinexor demonstrated a decrease in GBM tumor growth and prolonged animal survival in murine orthotopic patient-derived xenografts. [37]. Based on these preclinical results, many clinical trials are currently ongoing; specifically, two phase I recruiting trials in which Selinexor is used in combination with the standard therapy temozolomide and with radiotherapy, for newly diagnosed or recurrent glioblastoma. These studies may offer a new perspective that could change clinical practice in this kind of solid tumors.

### 3.7. Osteosarcoma

In osteosarcoma, roles of pediatric or medical oncology, surgery, and radiotherapy are equally primary and, in the last few years, researches have been investigating target therapy and immunotherapy [38,39]. A pre-clinical study, published in July 2021 by Moritz von Fallois et al. [40], described the role of Selinexor in human osteosarcoma cells and human hepatoma cells focusing on Hypoxia-inducible factor (HIF-1). Hypoxia is a favorable condition for tumors and also determines radioresistance. The authors measured HIF-1 levels in both cell lines, placed in both normoxic and hypoxic conditions, and subsequently administered Selinexor. The results showed that HIF-1 levels decrease especially in cell lines in hypoxic conditions, and, in particular, decrease by approximately 50% and 80% in human osteosarcoma cells and hepatoma cells, respectively. Moreover, the authors irradiated both cell lines with 2, 4, 6, or 8 Gray (Gy), after a treatment with Selinexor for 24 h, and there was a statistically significant radioresistance decrease. This last result demonstrates, for the first time, Selinexor’s role in reducing hypoxia pathogenetic properties, such us radioresistence.

## 4. Clinical Studies

### 4.1. Gynecologic Cancer

The early pre-clinical studies in ovarian cancer cells were instrumental in furthering the understanding of XPO1 inhibitors. XPO1 participates in the nuclear export of FOXO1, which decreases in platinum-resistant ovarian carcinoma. Ovarian cancer cells demonstrated sensitivity to the effects of Selinexor alone and in combination with Cisplatin. Selinexor significantly inhibits tumor growth, induces FOXO1 nuclear localization, and improves the efficacy of Cisplatin [41]. In the Phase II, open-label trial for advanced gynecologic malignancies, 114 patients, with ovarian (*n* = 66), endometrial (*n* = 23), or cervical (*n* = 25) cancer were enrolled and treated with selinexor (35 or 50 mg/m^2^ twice-weekly [BIW] or 50 mg/m^2^ once-weekly [QW]) in 4-week cycles. Disease Control Rate (DCR) was 30% (ovarian 30%; endometrial 35%; cervical 24%). Median Progression Free Survival (PFS) and Overall Survival (OS) for patients with ovarian, endometrial, and cervical cancer were 2.6, 2.8, and 1.4 months, and 7.3, 7.0, and 5.0 months, respectively. Considering all patients, nausea was observed in 67%, 73%, and 70%; fatigue 71%, 66%, and 75%; and weight loss 43%, 55%, and 30% (predominantly grade 1/2), in those treated with 35 mg/m^2^ BIW, 50 mg/m^2^ BIW, and 50 mg/m^2^ QW, respectively. In patients with ovarian cancer receiving 50 mg/m^2^ QW, less high-grade Adverse Events (AE) as well as a similar efficacy as BIW treatment were reported, which therefore should be preferred. These results showed Selinexor efficacy mostly in pretreated ovarian and endometrial cancers, and studies with Selinexor in combination with Cisplatin are ongoing [42].

### 4.2. Multiple Myeloma

Grosicki et al. evaluated the clinical benefit of weekly Selinexor plus Bortezomib, and dexamethasone versus Bortezomib and dexamethasone in patients who had previously been treated with one to three lines of therapy [28]. In this phase 3, randomized trial (BOSTON), 402 patients were randomized (1:1) to receive Selinexor (100 mg once per week), Bortezomib (1.3 mg/m^2^ once per week), and dexamethasone (20 mg twice per week), or Bortezomib (1.3 mg/m^2^ twice per week for the first 24 weeks and once per week thereafter) and dexamethasone (20 mg four times per week for the first 24 weeks and twice per week thereafter). Median progression-free survival (PFS) was 13.9 months versus 9.5 months with Selinexor, Bortezomib, and dexamethasone, and with Bortezomib and dexamethasone, respectively (hazard ratio 0.70 [95% CI 0.53–0.93], *p* = 0.0075). Anemia, fatigue, and thrombocytopenia were the most common adverse events; surprisingly, peripheral neuropathy was more frequent in the second group (Bortezomib and dexamethasone) than the first (Selinexor, Bortezomib and dexamethasone). This trial demonstrated that a once-per-week regimen of Selinexor, Bortezomib, and dexamethasone is a novel and effective treatment option for patients with multiple myeloma in progression after up to three previous lines of therapy.

### 4.3. Leukemia and Lymphoma

The activity of SINE compounds as treatment for acute leukemia has been studied extensively in pre-clinical and clinical trials. The nucleocytoplasmic dysregulation in acute myeloid leukemia (AML) was investigated by Kojima K. at al.; first of all, they demonstrated that elevated XPO1 levels are associated with poor prognosis and they explained the important role of mutated nucleophosmin (NPM1) and p53 in leukemogenesis. On one hand, this study demonstrated the important activity of SINEs, regardless NPM1 mutational status; on the other, it revealed that in AML, in the presence of a p53 mutation, SINEs’ activity was sensibly decreased. In this set of cell lines, a combination of SINEs and Nutlin-3a, as well a MDM2 inhibitor, could play a strategic role to induce cell apoptosis [43]. Recently, several phase I/II clinical trials emerged in which Selinexor was tested as a single-agent and in combination with various chemotherapy regimens for AML, which reported both tolerability and overall response rates ranging from 14–70% [44]. Like in acute leukemia, also in lymphomas the activity of SINE compounds is achieved through and related to the nuclear localization of p53, downregulation of c-myc and NFkB, and activation of different pathways leading to cell apoptosis [45]. A phase I clinical trial in patients with relapsed or refractory non-Hodgkin’s lymphoma found that Selinexor was well tolerated and resulted in an ORR of 31% [46]. Several early phase clinical trials evaluating the efficacy of SINE compounds in lymphoma are ongoing and their results are strongly expected.

## 5. Selinexor in Soft Tissue Sarcomas (STS). A Landscape on the Medical Approach

### 5.1. Chemotherapy

Soft tissue sarcomas (STS) account for only 1% of all solid malignant tumors. This large family of solid tumors derive from mesenchymal cells, commonly classified in about 50 subtypes; among others, these include: leiomyosarcoma, synovial sarcoma, liposarcoma, desmoid tumor, fibrosarcoma, vascular sarcoma, epithelioid sarcoma, and chondrosarcoma. Half of STS are a primary disease of one or more gastrointestinal organs, gynecological organs, urological organs, or head and neck [47]. Due to STS heterogeneity, both in histotype and origin sites, as well as their low incidence, it is difficult to develop clinical trials that focus on molecular-targeted therapy and, therefore, on new treatments for this type of cancer. For decades, Doxorubicin monotherapy has been the standard of care in metastatic STS, but there are specific subtypes sensitive to other antitumor agents. For example, synovial sarcoma, in which chromosomal translocation of t(X;18) (p11.2; q11.2) is a common characteristic [48], is sensitive to ifosfamide. Therefore, in the clinical management of patients with synovial sarcoma, ifosfamide-containing regimens are generally preferred. In a phase 2 trial, published in 2002, patients with leiomyosarcoma were treated with gemcitabine and docetaxel combination; there was a high response rate (53%) observed, in particular, in patients with uterine sarcoma [49]. For these good results, the combination of these drugs was introduced in clinical practice, although subsequent randomized studies did not show promising results. Paclitaxel is another anticancer agent used, both alone and in combination, to treat many solid tumors; paclitaxel was tested especially in cutaneous angiosarcoma, in fact, this type of STS develops mainly in elderly patients, therefore, its good tolerability combined with its low cardiotoxicity, leads experts to use it. One of the most important trials conducted on metastatic STS was EORTC 62012: 455 pts from 38 centers were randomized to Doxorubicin (*n* = 228) or Doxorubicin-Ifosfamide (D-I) (*n* = 227), with a median follow-up of 56 months. Overall Survival (OS) at 1 year was slightly greater with D-I but the difference was not statistically significant (HR 0.82, 95.5% CI 0.66–1.01). The lack of a significant improvement in OS does not support the routine use of this intensive combination of D-I for STS in the palliative setting [47]. So, until now, doxorubicin remains the gold standard in comparative first line studies for metastatic STS.

### 5.2. Targeted Therapy

One of the best examples of molecular targeted drugs recently approved for STS is Imatinib; this drug is a tyrosine kinase inhibitor (TKI) targeting c-KIT specifically expressed in gastrointestinal stromal tumors (GISTs) [50]. GIST is a mesenchymal tumor that originates from submucosal tissues in the gastrointestinal tract. Considering the excellent results of this targeted therapy, treatment strategies of GIST became different from those of other STS subtypes. Following Imatinib, new TKIs such as Sunitinib and Regorafenib were investigated and approved to treat GISTs [51,52]. Another successfully targeted therapy is Pazopanib, which is an oral, multi-target tyrosine kinase inhibitor (TKI) of platelet-derived growth factor (PDGFR) -α and -β; vascular endothelial growth factor receptors (VEGF) 1, 2 and 3; and stem cell factor receptor (c-KIT) [53]. The results of the phase III PALETTE trial, in which 369 patients with metastatic STS were randomly assigned to receive Pazopanib (*n* = 246) or placebo (*n* = 123), demonstrated a prolongation of median progression-free survival in patients who received Pazopanib (4.6 months vs. 1.6 months [HR] 0.31, 95% CI 0.24–0.40). Overall survival was 12.5 months (10.6–14.8) with Pazopanib versus 10.7 months (8.7–12.8) with placebo (HR 0.86, 0.67–1.11). Unfortunately, results showed heterogeneous ORR between the different sarcoma histotypes. For this reason, Pazopanib is approved as a treatment for patients with metastatic non-adipocytic STS who progressed to chemotherapy [54]. Conversely, Olaratumab, a recombinant human immunoglobulin G subclass 1 (IgG1) monoclonal antibody that specifically binds PDGFRα, in combination with Doxorubicin, failed to demonstrate an OS advantage over Doxorubicin alone (total STS: hazard ratio, 1.05 [95% CI, 0.84–1.30], *p* = 0.69, median overall survival, 20.4 months vs. 19.7 months) [55].

## 6. Selinexor: Preliminary Results as STS Treatment

In 2016, Nakayama et al. published an important preclinical study showing sarcoma cells sensibility to Selinexor [56]. The efficacy of Selinexor was investigated in-vitro and in-vivo using nine sarcoma xenograft models including gastrointestinal stromal tumor (GIST), liposarcoma, leiomyosarcoma, and undifferentiated sarcomas. In particular, in de-differentiated liposarcoma (LPS), Nakayama et al. [56] hypothesized that Selinexor might improve p53 and p21 activity by maintaining their nuclear localization. Results showed an increase in p53 and p21 protein expression. Moreover, p53 expression increased more significantly in the nucleus than in the cytoplasm, and phosphorylation of RB decreased following exposure to Selinexor. In this study, the authors analyzed also p53 mutant LPS line; they observed that Selinexor can induce apoptosis and G1-arrest in LPS cells regardless of p53 mutational status. Considering the great results of Selinexor in the hematological field, and encouraged by preclinical studies on sarcoma, a phase II study was designed by Gunder M. et al. to assess the efficacy and safety of Selinexor in advanced de-differentiated liposarcoma (SEAL trial) [57]. Fifty-six patients were randomized 1:1 to receive Selinexor (60 mg) or placebo twice weekly in 42 days cycle until PD or intolerability. Progression free survival (PFS) was established as a primary endpoint. This study showed an improvement of median PFS with Selinexor (5.6 months vs. 1.8 months, hazard ratio of 0.64). This result made it possible to continue the study and therefore move on to phase 3, which enrolled approximately 285 patients and randomized them 2:1. Notably, the trial allowed patients on placebo with objective progression to cross over to the Selinexor treatment arm. Of those who received Selinexor, there was a trend towards an improvement in the median overall survival compared to patients who started the trial on the placebo arm of the study and never crossed over to the Selinexor treatment arm. Phase 3 is ongoing and the estimated study completion date is December 2021. In Table 1, we have shown a summary of clinical studies with Selinexor. In Table 2 we have shown a list of registered clinical trials involving Selinexor in various rare cancers.

## 7. Conclusions

Cancer is a degenerative disease characterized by several common traits acquired via distinct mechanisms and at various times during the multistep process of tumor development. Nucleocytoplasmic transport has recently gained relevance because of the link between its dysregulation and onco-proliferative processes involved in non-hematological and hematological malignancies. SINE compound, including Selinexor, showed high efficacy in inhibiting cancer cell proliferation and inducing apoptosis, as reported by many preclinical in-vitro and in-vivo studies described in this review. The efficacy of Selinexor was also pointed out by several clinical studies where the clinical benefits, in terms of progression free survival and overall survival, were especially achieved for hematological malignancies. Selinexor could also be a valuable option in the treatment of rare and genetically heterogeneous tumors such as sarcomas, especially those that are no longer responsive to standard therapies tumors. Although the role of Selinexor in the treatment of sarcomas is still in an early stage, the promising results obtained foster the hope that one day it will become a concrete option for the treatment of patients with sarcoma.

## Figures and Tables

**Table 1:** Summary of clinical studies with Selinexor.

Authors	NCT Number	Diseases	Drug(s)	No. of Patients	Primary Endpoint	Results	Study Phase
Abdul Razak AR et al. [27]	NCT01607905	Advanced solid tumors	Selinexor	189	Safety and maximum—tolerated dose	35 mg/m^2^ twice a week	I
Grosicki S et al. [28]	NCT03110562	Multiple myeloma	Selinexor +bortezo- Mib +desameta-sone	457	PFS	13.93 vs. 9.46 months (HR 0.7 *p* = 0.0075)	III
Vergote IB et al. [42]	NCT02025985	Advanced gynaecologic malignances	Selinexor	114	DCR	Ovarian 30% Endometrial 35% Cervical 24%	II
Kuruvilla J et al. [46]	NCT01607892	Relapse or refractory non-Hodgkin Lymphoma	Selinexor	79	ORR	31%	I
Gounder MM et al. [57]	NCT02606461	Advanced unresectable dedifferentiated liposarcoma	Selinexor	56	PFS	5.6 vs. 1.8 (HR 0.64 *p* = 0.21)	II/III

**Table 2:** List of registered clinical trials involving Selinexor in various rare cancers.

NCT Number	Study Title	Status
NCT04811196	A study of different dosing schedules of Selinexor in sarcoma patients.	Recruiting
NCT03193437	Selinexor in patients with advanced thymic epithelial tumor progressing after primary chemotherapy.	Recruiting
NCT04138381	Selinexor as a single agent and in combination with Imatinib in patients with metastatic and/or unresectable GIST.	Recruiting
NCT04595994	Selinexor plus gemcitabine in selected advanced soft-tissue sarcoma and osteosarcoma.	Recruiting
NCT03042819	Study of Selinexor and Doxorubicin in advanced soft tissue sarcomas.	Active, not recruiting
NCT02323880	Selinexor in treating younger patients with recurrent or refractory solid tumors or high-grade gliomas.	Recruiting
NCT01986348	A Phase 2 Study evaluating the efficacy and safety of Selinexor in patients with recurrent gliomas	Completed
NCT01896505	A Phase I Trial to assess the effects of Food and Formulation on PK of KPT-330 in patients with sarcoma.	Completed

## Data Availability

Not applicable.
